# Enzymatic Switching Between Archaeal DNA Polymerases Facilitates Abasic Site Bypass

**DOI:** 10.3389/fmicb.2021.802670

**Published:** 2021-12-20

**Authors:** Xu Feng, Baochang Zhang, Ruyi Xu, Zhe Gao, Xiaotong Liu, Guanhua Yuan, Sonoko Ishino, Mingxia Feng, Yulong Shen, Yoshizumi Ishino, Qunxin She

**Affiliations:** ^1^CRISPR and Archaea Biology Research Center, Microbial Technology Institute and State Key Laboratory of Microbial Technology, Shandong University, Qingdao, China; ^2^Department of Bioscience and Biotechnology, Graduate School of Bioresource and Bioenvironmental Sciences, Kyushu University, Fukuoka, Japan

**Keywords:** translesion DNA synthesis, DNA polymerase, abasic site, Dpo2, Dpo4, enzyme kinetics, archaea

## Abstract

Abasic sites are among the most abundant DNA lesions encountered by cells. Their replication requires actions of specialized DNA polymerases. Herein, two archaeal specialized DNA polymerases were examined for their capability to perform translesion DNA synthesis (TLS) on the lesion, including *Sulfolobuss islandicus* Dpo2 of B-family, and Dpo4 of Y-family. We found neither Dpo2 nor Dpo4 is efficient to complete abasic sites bypass alone, but their sequential actions promote lesion bypass. Enzyme kinetics studies further revealed that the Dpo4’s activity is significantly inhibited at +1 to +3 site past the lesion, at which Dpo2 efficiently extends the primer termini. Furthermore, their activities are inhibited upon synthesis of 5–6 nt TLS patches. Once handed over to Dpo1, these substrates basically inactivate its exonuclease, enabling the transition from proofreading to polymerization of the replicase. Collectively, by functioning as an “extender” to catalyze further DNA synthesis past the lesion, Dpo2 bridges the activity gap between Dpo4 and Dpo1 in the archaeal TLS process, thus achieving more efficient lesion bypass.

## Introduction

Genomes in all forms of life are consistently insulted by endogenous and external sources of DNA damage agents. Most of the resulting genomic DNA damage is repaired by diverse DNA repair pathways that are evolutionarily conserved. However, upon extensive DNA damage exposure, DNA lesions can escape routine DNA damage repair mechanisms. These residual unrepaired DNA lesions can effectively hinder the progression of replicative DNA polymerase during genomic DNA replication, leading to more severe types of damage if not repaired in a timely fashion. Numerous studies have revealed that DNA polymerases specialized for replication of lesion-containing DNA templates are at play in dealing with those replication-blocking lesions and these specialized DNA polymerases are capable of bypassing DNA lesions in a process named translesion DNA synthesis (Friedberg et al., 2002). So far, most of these TLS polymerases are of Y-family. Structural analyses of PolY enzymes have revealed the mechanism of damage tolerance since they are characterized with a spacious active center that can accommodate DNA lesions (Sale, 2013). On the other hand, although many B-family DNA polymerases function as the replicase in eukaryotes and many archaeal species, some PolB enzymes are specialized in DNA repair such as the eukaryotic Pol ζ and bacterial Pol II (Yang and Gao, 2018).

Interestingly, PolY and PolB are found to work in concert in a two-polymerase mechanism in the eukaryotic TLS process. The mechanism starts with a Y-family polymerase that primarily inserts a nucleotide across the DNA lesion (inserter polymerase), generating a mispaired primer end, which is subsequently extended by Pol ζ, the specialized PolB that functions as an extender polymerase (Johnson et al., 2000; Haracska et al., 2001; Prakash and Prakash, 2002; Livneh et al., 2010). However, while the two-polymerase TLS process provides an important mechanism for DNA lesion bypass in Eukarya, the mechanism is absent from Bacteria. In the latter, both TLS insertion and extension are believed to be accomplished by the same TLS polymerase (Fujii and Fuchs, 2020). For example, the *Escherichia coli* Pol V of the Y-family is the main contributor to the DNA damage-induced mutagenesis, and the bacterial enzyme is competent both in the nucleotide insertion opposite the lesion and in the ensuing primer extension past the lesion site (Tang et al., 2000; Fuchs and Fujii, 2013; Goodman and Woodgate, 2013).

In the third domain of life, Archaea, Dpo4 and its homologues, belonging to the Y-family are well known as TLS polymerases. For example, *Saccharolobus solfataricus* Dpo4 (*Sso*Dpo4) has been characterized in great detail and has been used as a model to study the mechanism of lesion tolerance by DNA polymerases, and this has revealed that the polymerase is capable of bypassing different types of DNA lesion with varying efficiencies (Boudsocq et al., 2001; Rechkoblit et al., 2006; Fiala et al., 2007). Intriguingly, when tested for the bypass of an abasic site (apurinic/apyrimidinic, AP), one of the most abundant naturally occurring DNA lesions (Lindahl and Nyberg, 1972; Friedberg et al., 2005), *Sso*Dpo4 was inhibited not only in the nucleotide incorporation opposite the lesion, but also in the subsequent extension downstream of the lesion (Fiala et al., 2007). This suggests further extension past the lesion possibly requires the action of another DNA polymerase.

Organisms of Sulfolobales that thrive in acidic hot springs encode 4 DNA polymerases: Dpo1, -2, -3, and -4 among which Dpo4 is a PolY whereas the remaining 3 belong to the B-family (She et al., 2001; Chen et al., 2005; Guo et al., 2011). All 3 PolBs have been characterized. The replicative polymerase, Dpo1, exhibits strong exonuclease activity upon encountering the AP site (Gruz et al., 2003). Dpo3, another PolB that may also be involved in chromosome replication, also shows the 3′–5′ exonuclease activity (Choi et al., 2011; Bauer et al., 2012). In contrast, Dpo2, the third PolB that mediates the targeted mutagenesis in *Sulfolobuss islandicus* (Feng et al., 2020), is devoid of the 3′–5′ exonuclease activity (Feng et al., 2021). Nevertheless, it is still elusive whether these DNA polymerases could coordinate with each other to efficiently bypass DNA lesions, and how Dpo2, the only DNA damage-inducible DNA polymerase in the organism, could contribute to the DNA lesion bypass.

To study that, recombinant proteins of *S. islandicus* Dpo2 were obtained from the native host and investigated for its roles in AP bypass, along with homologously expressed Dpo4 and Dpo1 originated from the same organism. We show here that Dpo2 works in concert with Dpo4 to generate DNA patches around DNA lesions. The joint action of these two polymerases provides DNA substrates suitable for Dpo1 to resume chromosome replication. At last, a model is presented for the Dpo2-facilitated TLS pathway, involving multiple enzymatic switching.

## Materials and Methods

### *Sulfolobus* Strains and Cell Growth

*Sulfolobuss islandicus* E233S (Δ*pyrEF*Δ*lacS*), a derivative strain of *S. islandicus* Rey15A strain (Deng et al., 2009) was used as the host for recombinant protein expression. *Sulfolobus* strains were grown in SCV (0.2% sucrose, 0.2% casamino acids, 1% vitamin solution plus basic salts) or ACV media (0.2% D-arabinose, 0.2% casamino acids, 1% vitamin solution plus basic salts) at 78°C as previously described (Peng et al., 2012). The Dpo2 (SiRe_0615) (Feng et al., 2020) and Dpo1 (SiRe_1451) expression strain (Feng et al., 2021) were constructed previously and the Dpo4 expression strain was constructed in this study.

### Expression and Purification of Recombinant DNA Polymerases From *S. islandicus*

The Dpo2 and Dpo1 DNA polymerases were purified previously as described (Feng et al., 2021). The Dpo4-encoding gene (SiRe_0236) was amplified from *S. islandicus* genomic DNA using primers indicated in Supplementary Table 1 and cloned into the expression vector, pSeSD (Peng et al., 2012), using Nde I and Sal I restriction sites. Briefly, 20–500 ng expression plasmids were transformed to the E233S competent cells and positive colonies harboring the expressing plasmid were used for recombinant protein production. The colonies were first grown in SCV media, then inoculated into 11 L ACV media for protein induction. Cell mass harvested from the ACV cultures were used for the purification of Dpo4 by the following procedures. Cell pellets were resuspended in Buffer A (50 mM Tris–HCl, 200 mM NaCl, 30 mM Imidazole, pH 7.5) supplemented with 1× protease inhibitor cocktails and 10 μg/ml DNase I. Cell lysates were obtained by passing the cell suspension through a high-pressure homogenizer (JNBio). Cell debris in the lysates were removed by centrifugation at 15000 × *g* for 40 min and the supernatant was filtered through a 0.45 μm filter. The clarified supernatant was then applied to a Histrap HP column (Cytiva) and the bound proteins were eluted by Buffer B (50 mM Tris–HCl, 200 mM NaCl, 500 mM Imidazole, pH 7.5). Fractions eluted from the Histrap HP column were further purified by size exclusion chromatography with a Superdex 200 increase 10/300 GL column (Cytiva). Fractions containing Dpo4 of a high purity were pooled and concentrated using a 10K protein concentrator (Millipore). Concentrated proteins were preserved at −20°C in the presence of 50% glycerol. The concentration of each protein was determined using Bradford assay (Bradford, 1976), with BSA of known concentrations as standards. The purified archaeal polymerase was examined by SDS-PAGE and the result was shown in Supplementary Figure 1.

### DNA Substrates

All synthetic oligos including unlabeled, FAM-labeled primers, undamaged templates, and templates containing base modifications were synthesized and purified by HPLC at Genewiz (Suzhou, CN) or Sangon Biotech (Shanghai, CN). The sequence of primers and templates were listed in Supplementary Table 1. DNA substrates were prepared by annealing corresponding primer strand and template strand as indicated in Supplementary Table 2 at 1:1.5 ratio using a thermal cycler, in which the temperature was decreased by 0.2°C each cycle for 350 cycles after denaturation at 95°C for 5 min.

### Primer Extension Assay

Primer extension assay was set up in a 10 μl reaction containing 50 nM primer-template substrate, DNA polymerases with indicated concentrations, 100 μM dNTPs, 50 mM Tris–HCl (pH 8.0), 40 mM KCl, 10 mM MgCl_2_, and 0.1 mg/mL BSA. Reactions were incubated at 60°C for the time periods indicated in each experiment and terminated by addition of 10 μl 2× loading dye solution (1× TBE, 8 M Urea, 10 mM EDTA, 0.1% bromophenol blue), followed by denaturation at 95°C for 5 min and immediate chilling on ice. Replication products were resolved by Urea-18% PAGE and visualized by an Amersham ImageQuant 800 biomolecular imager (Cytiva).

### Single Nucleotide Incorporation Assay

The experiment was carried out as described for primer extension assay, except the dNTPs were replaced by 100 μM each dNTP in the reaction system.

### Steady-State Kinetics Analysis

Steady state kinetics was performed as described (O’Flaherty and Guengerich, 2014). To ensure that the reaction was in the linear range, products formation was kept to less than 20% of the starting substrate. Each 10 μl reaction contained either 11 nM Dpo2 or 2.5 nM Dpo4, 100 nM 5′ FAM-labelled substrate, a concentration range of dGTP, 50 mM Tris–HCl (pH 8.0), 40 mM KCl, 10 mM MgCl_2_, and 0.1 mg/mL BSA. The reaction was initiated by addition of dNTP with varying concentrations, and was terminated by mixing with 10 μl 2× loading dye solution (1× TBE, 8 M urea, 10 mM EDTA, and 0.03% bromophenol blue) and heating at 95°C for 5 min. Products were resolved in a Urea-18% PAGE gel and visualized by an Amersham ImageQuant 800 biomolecular imager. The percentage of products formation was quantitated using ImageQuant software and the velocity of dNTP incorporation was calculated by dividing the yield of products formed by the respective time of the reaction at each concentration of dNTP. The data was fitted into Michaelis-Menten equation using Graphpad prism software, from which the apparent *k*_cat_ and *K*_*m*_ values were determined. The substrates used in the kinetics assay include D1–D8, L1–L8, and S0, an undamaged DNA substrate control, as shown in Supplementary Table 2. The polymerization efficiency (*k*_eff_) of each enzyme on different AP-containing substrate with different TLS patch was expressed as: *k*_eff_ = *k*_cat_/*K*_*m*_ and the Relative *k*_eff_ was calculated by (*k*_cat_/*K*_*m*_)_*AP*_/(*k*_cat_/*K*_*m*_) _undamaged_.

### Two-Step DNA Polymerase Assay

Each 20 μl reaction mixture was prepared in microfuge tubes containing 50 nM L_*AT*_ substrate, a DNA polymerase or a combination of two DNA polymerases with indicated concentration(s) and 100 μM dNTPs in the same reaction buffer as for the primer extension assay. The difference was that each reaction in two-step experiments consisted of two sequential steps of primer extension. The first primer extension was conducted by incubating the reaction mixtures at 60°C for 5 min. Then, the first DNA polymerase in the reaction mix in each tube was inactivated by heating up to 98°C for 10 min. These microfuge tubes were allowed to cool down to the room temperature in 15 min. After bringing down all solution to the bottom of the tube by centrifugation at 18514 × *g* for 5 min, each sample was divided into two aliquots. One was for the second primer extension, and this was done by addition of another DNA polymerase to the reaction mix. The other was a reference, and for that purpose, only the enzyme dilution buffer was added to the tube. The second primer extension reaction was also conducted at 60°C for 5 min. In addition, one-step primer extension reactions were also conducted with each of Dpo2, Dpo4 and Dpo1 as well as their combinations and used as references. All primer extension products were analyzed by Urea-18% PAGE electrophoresis and visualized by an Amersham ImageQuant 800 biomolecular imager (Cytiva).

### Exonuclease-Polymerase Activity Transition Assay for Dpo1

Each 10 μl reaction contained 100 nM substrate from D1-D8 or L1-L8, 25 nM Dpo1, 100 μM dNTPs and the reaction buffer as used in the primer extension assay. The experiment was carried out at 60°C for 5 min.

### Image Processing and Data Fitting

Gel images were taken using an Amersham ImageQuant 800 biomolecular imager under an auto-exposure mode. The percentage of products formation or primers extended were then quantified using ImageQuant software by measuring the grey value of regions of products or primers. After subtracting the corresponding background value, the ratio of product formation or primer extended was calculated by dividing the value of product or primer by the value of (Product + Primer). For Kinetics analysis, the rate of product formation at each dNTP concentration was calculated individually and plotted against corresponding dNTP concentration. The data was fitted into Michaelis-Menten equation, from which apparent *K*_*m*_ and *k*_cat_ values were determined. The final value shown for each data point represent an average of triplicates ± SD.

## Results

### Dpo2 Elongates AP-Mispaired Primer Termini *via* a Lesion Loop-Out Mechanism

**FIGURE 1:**
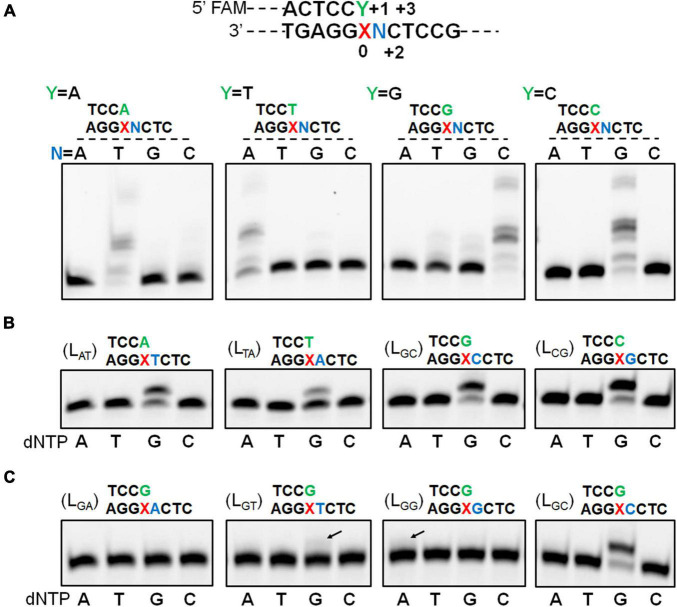
Dpo2 elongates AP-mispaired primer termini *via* a lesion loop-out mechanism. **(A)** TLS extension by Dpo2 exhibits DNA substrate preference. A general schematic of substrate is shown with the AP site and its immediately flanking positions are marked as “0” and “+1 to +3.” Sixteen different DNA substrates depicted in the upper part of the panel **(A)** were tested. Primer extension reactions were set up with 37.5 nM Dpo2, 50 nM substrate, and 100 μM dNTPs, and incubated at 60°C for 10 min. N represents one of the four possible nucleotides. **(B)** Dpo2 mediates TLS extension *via* a lesion loop-out mechanism. Four substrates (L_*AT*_, L_*TA*_, L_*GC*_, and L_*CG*_) were employed for the single nucleotide-incorporation experiment, which was conducted as described in panel **(A)** except for dNTPs, which were replaced with dATP (A), dTTP (T), dGTP (G), or dCTP (C) as indicated below the gel image. **(C)** Dpo2 is capable of TLS extension *via* an additional loop-out mechanism. The assay was performed as described in panel **(B)** using L_*GA*_, L_*GT*_, L_*GG*_, L_*GC*_ substrates.

We have recently found that *Sulfolobus* Dpo2, an exo^–^ B-family DNA polymerase, is very inefficient in nucleotide incorporation, but readily extends primer termini paired to the AP lesion (Supplementary Figure 2; Feng et al., 2021). A closer examination of replication products by Dpo2 on AP-containing templates revealed that the longest product is 1 nucleotide shorter than the template strand, indicative of the occurrence of a single nucleotide omission during the TLS extension (Supplementary Figure 2). To uncover the possible underlying mechanism, four DNA templates and four primers were synthesized (Supplementary Table 1). The 4 templates contained each of the 4 possible nucleotides at the +1 position immediately 5′-flanking the AP site whereas termini of the 4 primers extending to the position opposite to the AP site also ended up with all 4 possible nucleotides. As a result, annealing of individual template with each of the primers generated 16 different primer-template substrates (Supplementary Table 2). These DNA substrates were then employed to investigate the ability of Dpo2 to extend the primer termini past the AP site. As shown in Figure 1A, in the presence of four dNTPs, primer extension by Dpo2 showed substrate preference because the primer extension only occurred for the substrates in which the primer terminal base could form base-paring with the +1 template base. These results suggested that the lesion on the template could be looped out during the Dpo2 replication. To test that, the four “paired” substrates (L_*AT*_, L_*TA*_, L_*CG*_, and L_*GC*_) were selected and tested for the preference of nucleotide incorporation by Dpo2 in the presence of each dNTP. As shown in Figure 1B, only dG was incorporated on the four substrates, suggesting that the dC at +2 site could have served as the template for the incoming nucleotide. These results confirmed that Dpo2 mediates AP lesion bypass primarily *via* the lesion loop-out mechanism.

Noticeably, additional extension products appeared when a dG primer terminus was combined with the templates containing a dG or dT at the +1 position (Figure 1A). We then examined the nucleotide incorporation preference by Dpo2 on the four substrates (L_*GA*_, L_*GT*_, L_*GG*_, and L_*GC*_). As shown in Figure 1C, primer extension activity was robust when the 5′ adjacent template base was dC. Furthermore, a weak activity of dG insertion was observed when the 5′ base was a dT, suggesting the primer terminal base dG may also wobble basepair with the 5′ dT of the lesion, such that the downstream dC can serve as the template for the incoming dGTP. In addition, dA could also be incorporated in the presence of a template GCT sequence beyond the lesion. In this scenario, the primer terminal dG base may pair with template dC at +2 to yield a 2-nt loop-out, allowing dT at +3 position to guide the incorporation of incoming dATP. Alternatively, the primer terminal dCdG may pair with template dGdC at +1 and +2 to yield a 1-nt loop-out, allowing dT at +3 position to guide nucleotide incorporation.

### The Influences of the 5′ Base to the Lesion on the Nucleotide Insertion Activity of Dpo2 and Dpo4

**FIGURE 2:**
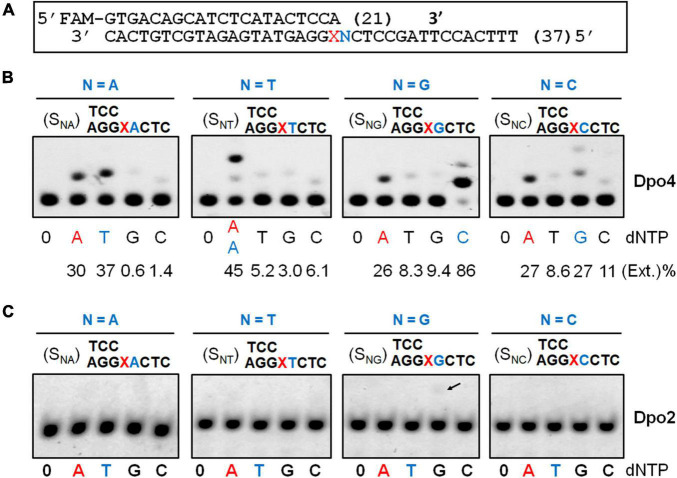
Nucleotide incorporation opposite the AP lesion by *Sis*Dpo4 and *Sis*Dpo2. **(A)** DNA substrates used in panels **(B,C)**. **(B)** Nucleotide incorporation opposite the AP lesion by *Sis*Dpo4. DNA substrates are shown above the corresponding gel images whereas dNTP used for testing the nucleotide incorporation are shown below the image along with efficiencies of the incorporation. Nucleotide incorporation was tested with 50 nM substrates, 100 μM of indicated dNTP, and 11 nM Dpo4. The nucleotide inserted under the “A” rule and the “5′ rule” (lesion loop-out) was highlighted in red and blue, respectively. **(C)** Nucleotide incorporation opposite the AP lesion by *Sis*Dpo2. The experiment was carried out as described for panel **(A)**, except 37.5 nM Dpo2 was used.

Previous studies showed that *S. solfataricus* Dpo4 expressed in an *E. coli* host utilizes dual modes of nucleotide incorporation upon encountering an AP site in the DNA template: It either inserts a dATP opposite the AP site (the “A rule”) and further extends the mispaired end in the “direct extension mechanism,” or uses the template base 5′ to AP to instruct the nucleotide incorporation (the “5′ rule”) in the “lesion loop-out mechanism” (Ling et al., 2004; Fiala and Suo, 2007). To test if the *S. islandicus* Dpo4 expressed from the native host (Supplementary Figure 2) could possess the same properties, a series of primer-template substrates were generated (Figure 2A), and tested for the effect of the 5′ base on the nucleotide incorporation preference of Dpo4 across the lesion. As shown in Figure 2B, the *S. islandicus* Dpo4 also adopted the “A rule” or the “5′ rule” for nucleotide incorporation at the lesion site, and the enzyme inserts dA opposite the lesion irrespective of a 5′ sequence of the lesion, which is consistent with the results obtained for *E. coli* expressed *S. solfataricus* Dpo4 (Ling et al., 2004). However, endogenously expressed Dpo4 preferably utilized the 5′ rule for the nucleotide incorporation when the 5′ base is a dG or dA, and it equally used the two mechanisms in the case of a 5′ dC.

In a previous work, we showed Dpo2 is very inefficient in nucleotide incorporation opposite the AP site under the tested condition (Feng et al., 2021). To gain a further insight into the properties of Dpo2, the same set of DNA substrates, which was used for testing Dpo4 AP insertion activity above, was employed in the assay. As shown in Figure 2C, Dpo2 failed to yield any detectable extended products regardless of the identity of the 5′ base to the lesion except for a faint band (indicated by an arrowhead in Figure 2C) produced in the presence of a dG 5′ to the lesion. We regarded the extension product was generated from the 2-nt lesion loop-out mechanism described above. This was because base pairing could occur between the 5′ template base dG (+1) and the primer terminal nucleotide dC at -1. Then, the next 5′ template base dC (+2) could guide the incoming dGTP for DNA synthesis to yield the observed product in Figure 2C. Thus, these results also supported the conclusion that Dpo2 is very inefficient in nucleotide incorporation opposite the AP site. Together, the complementary activities between these two enzymes at the lesion site suggest that the primer termini generated by Dpo4 at the lesion site might be the substrates for Dpo2.

### Dpo2 and Dpo4 Cooperate in AP Lesion Bypass

**FIGURE 3:**
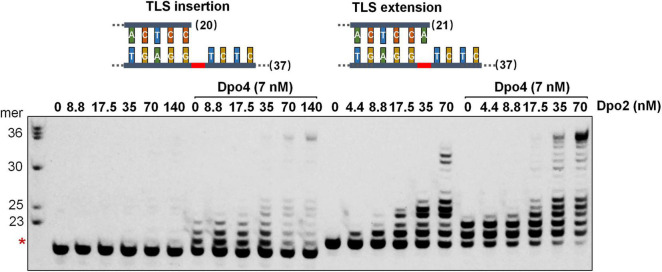
Synergetic DNA synthesis by Dpo2 and Dpo4 in AP bypass. A series of concentrations of Dpo2 alone, or in combination with 7 nM Dpo4, were tested for primer extension with 50 nM TLS insertion substrate (S_*NT*_) and TLS extension substrate (L_*AT*_) individually. The reaction was incubated at 60°C for 5 min. The red asterisk indicates the position of the AP lesion.

To investigate whether Dpo2 and Dpo4 could cooperate in AP lesion bypass, the two polymerases were tested for TLS insertion or TLS extension either alone or in combination. As shown in Figure 3, while Dpo2 failed to insert any dNTP opposite the lesion, the enzyme efficiently extended 3′-primer termini opposite to the lesion. In contrast, while Dpo4 incorporated 1–3 nucleotides around the lesion, the PolY enzyme was very inefficient in extending these replication intermediates since products of <+3 nucleotides past the lesion accumulated (Figure 3 and Supplementary Figure 3). In fact, neither Dpo2 nor Dpo4 alone produced full length products with these two substrates under the tested condition. However, in the presence of both polymerases, longer products near the full-length were synthesized with each substrate (Figure 3). These results support our hypothesis that Dpo4 is primarily responsible for the incorporation of nucleotides across the lesion, whereas Dpo2 primarily extends primers past lesion. To test whether Dpo2 and Dpo4 could also cooperate on undamaged template, we examined their activities on an undamaged substrate that has the same sequence as the AP-containing template except the lesion site. As shown in Supplementary Figure 4, the combined actions of Dpo2 and Dpo4 produced similar replication products, relative to Dpo4 alone. These results indicated the synergistic effect observed for these two enzymes with the AP-containing substrate was not resulted from the increase of the total enzyme concentration.

### Dpo2 and Dpo4 Act Sequentially Past AP Lesion

**FIGURE 4:**
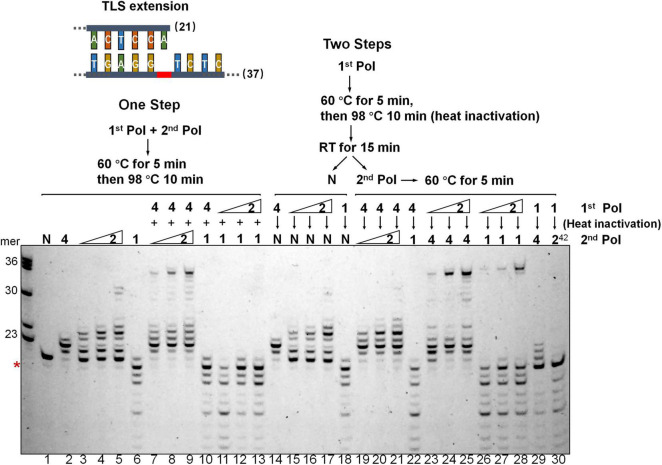
Dpo2 and Dpo4 act sequentially past AP site. Two-step polymerase assay of primer extension with Dpo2, Dpo1, and Dpo4 individually or in combinations. Schematic of both “One Step” and “Two Steps” assays are shown. 7 nM Dpo4 and 35 nM Dpo1 were used in the assays whereas Dpo2 used was 28, 35, or 42 nM. N denotes a negative reference lacking any polymerase enzyme. 1, 2, and 4 denote Dpo1, Dpo2, and Dpo4, respectively. 2^42^ means 42 nM Dpo2. The red asterisk indicates the position of the AP lesion.

To investigate if DNA synthesis past the AP lesion could involve any polymerase exchanges between Dpo2 and Dpo4, a two-step assay was employed in which only one polymerase was allowed to react at each step, either in the order from Dpo4 to Dpo2, or vice versa, using the TLS extension substrate shown in Figure 3. As shown in Figure 4, we found, only a few nucleotides were synthesized in the order of Dpo4-Dpo2 (Figure 4, lane 19-20-21), but longer DNA fragments close to the full-length were seen in the opposite order, i.e., from Dpo2 to Dpo4 (Figure 4, lane 23-24-25), which is very similar to the result seen in the reaction with simultaneous addition of Dpo2 and Dpo4 (Figure 4, lane 7-8-9) in the one-step reaction. These results suggest Dpo2 and Dpo4 act sequentially past the AP lesion and a polymerase exchange from Dpo2 to Dpo4 occurred in this process.

We then included Dpo1, the replicative polymerase, in the two-step assay, either in the combination of Dpo4-Dpo1 or Dpo2-Dpo1. Since Dpo1 could effectively degrade the primer termini, simultaneous addition of Dpo1 with either Dpo2 or Dpo4 did not yield any bypass products (Figure 4, lane 10-11-12-13), as would be expected. Strikingly, the sequential actions of Dpo2 and Dpo1 generated the longer replication products (Figure 4, lane 26-27-28). Thus, both pol combinations (Dpo2-Dpo4 and Dpo2-Dpo1) were able to bypass AP lesion more efficiently than Dpo4 alone or in the Dpo4-Dpo1 combination. Collectively, though Dpo4 is able to perform nucleotide insertion opposite the lesion, it is inefficient in extending from the inserted nucleotides. Dpo1 is also inefficient in the extension stage of lesion bypass due to its 3′–5′ proofreading activity, leaving an activity gap which would hamper efficient AP lesion bypass. Herein, these results indicated the action of Dpo2 may bridge the activity gap between Dpo4 and Dpo1, thus providing an alternative lesion-bypass pathway in archaea.

### Enzymatic Switching Between Archaeal Polymerases During the DNA Synthesis Past an AP Site

**FIGURE 5:**
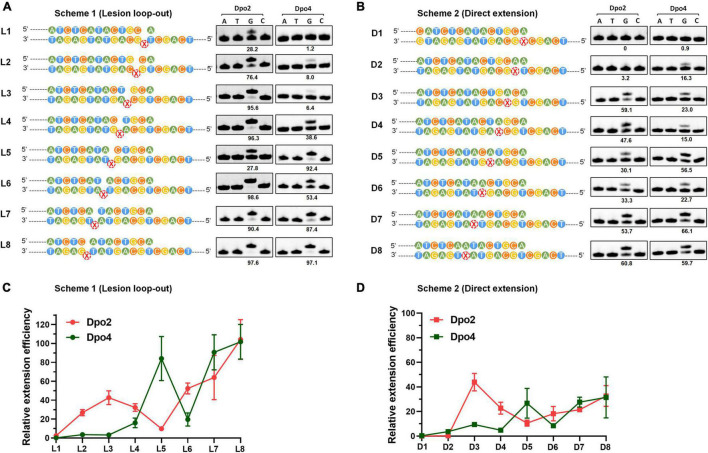
Enzymatic switching of archaeal polymerases past AP lesion. **(A)** Single nucleotide incorporation on AP-containing substrates by Dpo2 and Dpo4 following the lesion loop-out mechanism. Each reaction contains 37.5 nM Dpo2 or 2.7 nM Dpo4, 100 μM each dNTP and 100 nM substrate, and the experiment was carried out at 60°C for 3 min. X represents AP site. The extent of extension was evaluated by the percentage of primer extended (numbers below each lane). The sequence context of each template was indicated at the left of each panel. **(B)** Single nucleotide incorporation on AP-containing substrates by Dpo2 and Dpo4 following the direct extension mechanism. The experiment was carried out as above described. **(C)** The relative efficiency of primer extension by Dpo2 and Dpo4 *via* the lesion loop-out mechanism determined by steady-state kinetics, using substrates shown in Scheme 1. The efficiency of extending each substrate by Dpo2 or Dpo4 is defined by (*k*_cat_/*K*_*m*_)_*damaged*_/(*k*_cat_/*K*_*m*_)_*undamaged*_. The values are represented as mean ± SD. **(D)** The relative efficiency of AP bypass by Dpo2 and Dpo4 *via* direct extension mechanism determined by steady-state kinetics, using substrates shown in Scheme 2.

To gain a mechanistic insight into the polymerase exchange, two series of primer-template substrate were generated: one mimicking replication intermediates of the lesion loop-out mechanism (Scheme 1, Figure 5A), while the other, direct extension (Scheme 2, Figure 5B). These two sets of primer-template substrates were designed in which DNA templates were of a fixed length whereas the position of the AP site in them was changed in a step-by-step manner (Supplementary Table 1). These substrates were then used in the single nucleotide incorporation assay and in the steady state kinetics assay, to decipher how the activity of Dpo2 or Dpo4 could fluctuate at different positions around the lesion. As shown in Figures 5A,B, the dGTP was the only nucleotide incorporated in all the substrates, confirming that the primers and templates were paired as illustrated in the Scheme 1 and Scheme 2, respectively. In general, the primer was extended faster in the lesion loop-out mode than in the direct extension mode by both Dpo2 and Dpo4 (Figure 5), suggesting that the former is the preferable pathway to be used by both enzymes under the tested condition. Interestingly, we noticed the activity of Dpo4 and Dpo2 decreased at +6 and +5 site, respectively (Figure 5A), indicating these sites may represent inhibitions sites for these two TLS polymerases.

Next, we employed the steady state kinetic assay to quantitively determine the extension efficiencies of Dpo2 and Dpo4 from these two sets of substrates. The resulting *K*_*m*_ and *k*_cat_ values were summarized in Supplementary Table 3. Their stepwise change of the extension efficiencies following the lesion loop-out mechanism was illustrated in Figure 5C, in which Dpo2 exhibits low *K*_*m*_ values when extending primer termini positioned from +2 to +4. This suggests that the AP site in these substrates can be accommodated by Dpo2 in such a fashion that the lesion does not significantly impair the geometry of reactants. At +5 site, however, Dpo2 exhibited a reduced turnover number (*k*_cat_) and an increased *K*_*m*_ value, indicative of an unfavorable position of the primer terminus for the Dpo2 DNA synthesis. Very similar results were obtained in the extension experiments with the direct extension substrates (Figure 5D and Supplementary Table 3). These results further demonstrate that the +5 position constitutes a kinetically inhibitory site for Dpo2 for further TLS extension, and DNA synthesis at this position is very likely to be replaced by another DNA polymerase.

On the other hand, kinetic analysis with Dpo4 on these substrates showed that the PolY suffers from kinetic inhibition at +1, +2, +3, and +4 site in both schemes (Figures 5C,D and Supplementary Table 3). Interestingly, the inhibition is relieved at +5 position, before shallowing down again at +6. Thereafter, DNA synthesis by Dpo4 gradually resumes (Figures 5C,D). Since the activities of Dpo2 and Dpo4 are fully complementary to each other in DNA synthesis at the positions across and past the AP site, this strongly suggested that the two enzymes could act together *in vivo* for efficient AP bypass.

### Positions +5/6 Past the Lesion Constitute the Transition Site for the Exonuclease and Polymerase Activities of the Dpo1 Replicase

**FIGURE 6:**
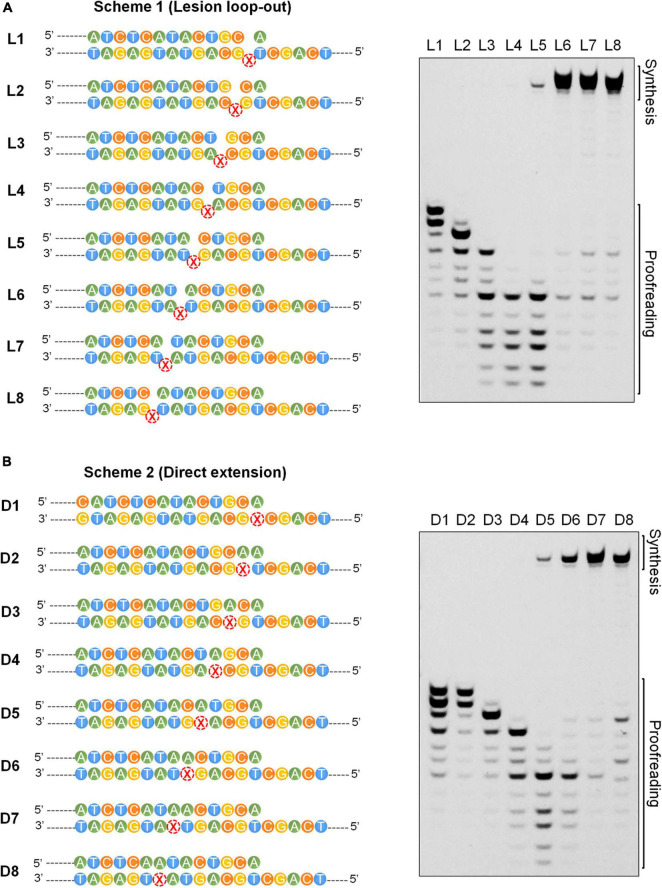
Effects of the length of TLS patch on the activities of Dpo1. **(A)** Transition from exonuclease activities to polymerase activities in Dpo1 with the increase of the length of TLS patches under the lesion loop-out mode. Each reaction contained 25 nM Dpo1, 100 μM dNTPs and 100 nM substrate, and was incubated at 60°C for 5 min. **(B)** Transition from exonuclease activities to polymerase activities in Dpo1 with the increase of the length of TLS patches under the direct extension mode. The assay was carried out as described for panel **(A)**.

The final step in the AP lesion bypass is to produce TLS DNA patches that have overcome the Dpo1 exonucleolytic degradation barrier and hand them back to the replicase. To reveal the minimal size of TLS patches required for efficient elongation by Dpo1 post an abasic site, we performed primer extension assays for Dpo1 using L1–L8 and D1–D8 substrates individually. As shown in Figures 6A,B, Dpo1 exhibited a strong exonuclease activity on substrates carrying a primer terminus ended at a position <+5. However, a sharp transition occurred from the exonuclease activity to the DNA polymerase activity of the Dpo1 replicase with the substrates carrying the primer terminus of +5 relative to those carrying +6 primer terminus. Conceivably, Dpo1 takes over the primer termini at +5 or +6 position past the lesion and resumes the normal DNA synthesis. Together, these results suggested that the TLS patches produced by Dpo2 alone, or by the joint actions of Dpo2 and Dpo4 are sufficient for Dpo1 to efficiently resume the chromosome replication.

## Discussion

**FIGURE 7:**
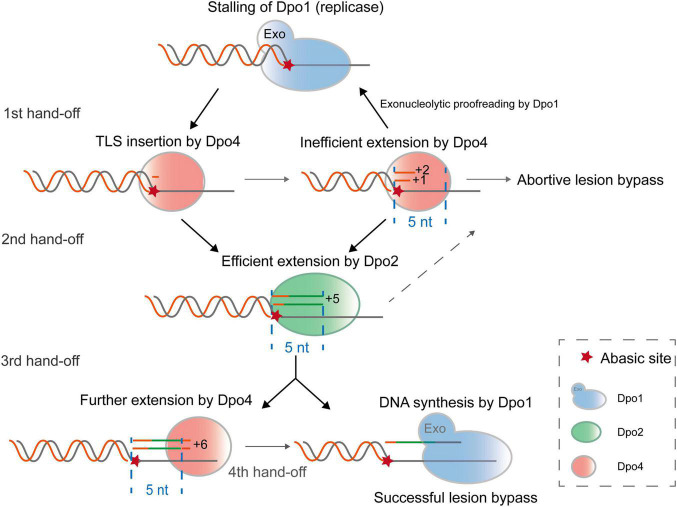
A proposed model for Dpo2-facilitated translesion DNA synthesis. Upon encountering a replication-blocking lesion in *S. islandicus*, the main replicase Dpo1 stalls and dissociates from the primer terminus preceding the lesion. The vacant primer terminus can be accessed by different polymerases but Dpo4 shows a high propensity to incorporate a nucleotide opposite the lesion. Since Dpo4 is intrinsically inefficient in extending primer matched to the lesion (position 0), +1 and +2 position, the primer termini containing the lesion can be handed over to Dpo2, which efficiently extends few nucleotides and reaches +5, an intrinsically inefficient site for this extender polymerase. At this point, Dpo1 or Dpo4 probably take over the DNA synthesis and in the latter case, the activity of Dpo4 is to be inhibited at +6, a next intrinsically inefficient position. As a result, Dpo4 and Dpo2 collectively generate a 5–6 nt TLS patch, which can be extended efficiently by the replicative polymerase Dpo1.

In this study, we biochemically characterized three distinctive archaeal DNA polymerases of *S. islandicus* and found that Dpo2, a B-family DNA polymerase, and a Y-family Dpo4, function synergistically in the archaeal TLS. The joint actions of these two polymerases generates short lesion-containing DNA patches that overcome the exonuclease activity of the replicase (Dpo1), thus achieving more efficient lesion bypass than one polymerase alone (Figure 7). In conclusion, by functioning in the extension step of lesion bypass, Dpo2 bridges the activity gap between the Dpo4 and the replicase to complete the lesion bypass process.

*Sso*Dpo4 is one of the TLS polymerases that have been best characterized *in vitro*. Several studies have demonstrated that it manifests TLS incorporation opposite a myriad of different DNA lesions, however, its efficiency in translesion synthesis can be very different upon encounter of different lesions (Boudsocq et al., 2001; Ling et al., 2004; Rechkoblit et al., 2006; Fiala and Suo, 2007). This is well exemplified with its capability in mismatch extension and the extension step during lesion bypass. For example, the archaeal PolY is inefficient in extending mismatched base pairs, and this is also applied to the extension of G:T base pair even though the wobble base pairing has a similar geometry as the Watson-Crick base pair (Trincao et al., 2004). In fact, many common DNA lesions have imposed a kinetic inhibition to the TLS extension by this archaeal PolY, such as the 6-4 photoproducts generated by UV irradiation (Boudsocq et al., 2001), 8-oxodG sites (Cranford et al., 2020), as well as AP sites observed in this study and in a previous work (Fiala et al., 2007). In addition, upon DNA damage treatment, the diversity and quantity of genomic DNA lesions in *Sulfolobus* cells are expected to be increased, but the cellular content of Dpo4 protein remains constant since its expression is not DNA damage-inducible (Feng et al., 2020). For these reasons, there is a great need for the archaeal PolY to function in concert with another DNA polymerase with a complementary kinetic feature to facilitate the lesion bypass in this organism. But such a DNA polymerase has been missing in *Sulfolobales* species since all remaining 3 DNA polymerases in *S. solfataricus* have been characterized and they show the 3′–5′ exonuclease activity. As a consequence, none of them are compatible with the requirement of the specialized extender DNA polymerase. However, by expression of *S. islandicus* DNA polymerases in their native host and characterization of the purified enzymes, we recently found that the *S. islandicus* Dpo2 is proficient in mismatch and TLS extension, and it is devoid of any exonuclease activity, thereby does not perform proofreading (24). Herein we further showed that Dpo2 and Dpo4 have complementary DNA synthesis capabilities and propose that these two TLS polymerases work in concert in translesion synthesis to effectively bypass DNA lesions in *S. islandicus*. Such system thus represents a strong candidate for the prokaryotic two-polymerase TLS pathway. Considering the two DNA polymerases have to deal with a large spectrum of DNA lesions upon an exposure of intensive DNA damage, we regard invoking the two-polymerase mechanism to increase the efficiency of DNA lesion bypass would constitute the first priority for the stressed archaeal cells. Taken together, the available data strongly support that Dpo4 enzyme functions as an inserter polymerase and acts together with Dpo2, an extender DNA polymerase, to facilitate translesion DNA synthesis in Archaea.

The combined actions of Dpo2 and Dpo4 in the translesion bypass of abasic sites may give rise to distinctive mutations, including frameshifts and base substitutions both of which can be explained by the lesion bypass mechanisms revealed in this work. The frameshift mutations could be generated from the lesion loop-out mechanism whereas the base substitution mutations could be yielded from the direct extension mechanism. The effect of DNA polymerases on the generation of the spectrum of spontaneous mutations in crenarchaea has been investigated by gene knockout in *S. islandicus* and *S. acidocaldarius*. For instance, genetic analyses of the *S. islandicus dpo4* and *dbh*, its *S. acidocaldarius* homolog, revealed that these PolYs facilitate frameshift mutations during the normal cell growth (Sakofsky et al., 2012; Feng et al., 2020). Interestingly, upon DNA damage treatment with NQO or UV, the mutation rate increased dramatically in *S. islandicus* and the effect is Dpo2-dependent, and these results indicated that the PolB2 enzyme is responsible for the elevated rate of mutation (Feng et al., 2020). Since the majority of NQO/UV-induced mutations are G to A and C to T transitions, the *in vivo* DNA damage repair by Dpo2 should be strongly biased to the direct extension mechanism to yield point mutations from these DNA damage treatments. Furthermore, the *in vivo* Dpo2 activity could be modulated by replication-associated factors, other DNA polymerases and/or Dpo2-associated factors as suggested previously (Feng et al., 2020), and investigation of their interactions would reveal additional TLS mechanisms.

While the results reported in this work do not provide a full picture of the *in vivo* mechanism of polymerase switching because of the lack of inclusion of the processivity factor PCNA, the replication processivity factor (Dionne et al., 2003; Xing et al., 2009), we are able to infer possible primer hand-off positions in the process of AP lesion bypass by three *S. islandicus* DNA polymerases, based on the kinetic properties of each DNA polymerase elucidated in this work. These include the recruitment of Dpo4 for nucleotide insertion across the lesion (0 position), the primer handoff from Dpo4 to Dpo2 at +1/+2, and backhanding of the primer from Dpo2 to Dpo4 at +5, as well as the transfer of primer to Dpo1, the high-fidelity replicase, at +6 past the lesion (Figure 7). Several lines of evidence support the hypothesis. For the first primer handoff, several studies have implicated Dpo4 for TLS insertion at AP site (Boudsocq et al., 2001; Ling et al., 2004; Fiala et al., 2007) and this is consistent with the flexible active center of PolY that can accommodate base pairing of damaged bases (Ling et al., 2001, 2004). Furthermore, both Dpo4 and Dpo1 interact with PCNA, suggesting that the sliding clamp may facilitate the polymerase switch from Dpo1 to Dpo4 at stalled replication forks. For the second primer handoff from Dpo4 to Dpo2, the necessity has been inferred not only from their complementary kinetic features in AP lesion bypass, but also from their gene deletion analysis in *S. islandicus*, where all targeted mutations are Dpo2-dependent (Feng et al., 2020). Theoretically the third switch can occur either from Dpo2 to Dpo1 or from Dpo2 to Dpo4 at +5/+6 sites. Strikingly, the minimal DNA patch (+5/6) that can be extended by the archaeal replicase is consistent with the results obtained for all tested replicases including bacterial Pol III (Kiefer et al., 1998; Fujii and Fuchs, 2004) and eukaryotic replicases (Zhang et al., 2002; McCulloch et al., 2004). Intriguingly, a 5–6 nt TLS patch corresponds to the length of a half turn of B-form DNA helix, possibly the looped-out AP lesion was presented in unfavorable positions for these archaeal polymerases, therefore interfering further catalysis. It would be very interesting to study how the AP lesion being accommodated by the DNA polymerases at the TLS extension stages, and if the similar pathway as proposed for *S. islandicus* could also operate in other organisms.

## Data Availability Statement

The original contributions presented in the study are included in the article/Supplementary Material, further inquiries can be directed to the corresponding author/s.

## Author Contributions

QS and XF designed the work and wrote the manuscript. XF, BZ, ZG, RX, XL, MF, SI, YS, YI, and QS contributed to the acquisition and analysis of the data. XF, BZ, QS, SI, YI, and YS interpreted the data. QS, XF, SI, YI, and YS revised the manuscript. All authors approved the final version of the manuscript.

## Conflict of Interest

The authors declare that the research was conducted in the absence of any commercial or financial relationships that could be construed as a potential conflict of interest.

## Publisher’s Note

All claims expressed in this article are solely those of the authors and do not necessarily represent those of their affiliated organizations, or those of the publisher, the editors and the reviewers. Any product that may be evaluated in this article, or claim that may be made by its manufacturer, is not guaranteed or endorsed by the publisher.
