# Supplementary material for: Enzymatic Switching Between Archaeal DNA Polymerases Facilitates Abasic Site Bypass
Source: Front Microbiol. 2021 Dec 20;12:802670. doi: 10.3389/fmicb.2021.802670 (PMC8721586; doi:10.3389/fmicb.2021.802670)
Supplement: Supplementary file 1 [file Data_Sheet_1.pdf]

Supplementary Material

Supplementary Tables

Supplementary Table S1. Oligonucleotides Used in This Study

**Primer strand**	**Sequence (5' to 3')**	**Source**
P2	5' FAM-GTGACAGCATCTCATACTCC	Genewiz
P3	5' FAM-GTGACAGCATCTCATACTCCA	Genewiz
P3T	5' FAM-GTGACAGCATCTCATACTCCT	Genewiz
P3G	5' FAM-GTGACAGCATCTCATACTCCG	Genewiz
P3C	5' FAM-GTGACAGCATCTCATACTCCC	Genewiz
pL0	5' FAM-GTGACAGCATCTCATACTGCA	Sangon
pD1	5' FAM-GTGACAGCATCTCATACTGCAA	Sangon
pD2	5' FAM-GTGACAGCATCTCATACTGACA	Sangon
pD3	5' FAM-GTGACAGCATCTCATACTAGCA	Sangon
pD4	5' FAM-GTGACAGCATCTCATACATGCA	Sangon
pD5	5' FAM-GTGACAGCATCTCATAACTGCA	Sangon
pD6	5' FAM-GTGACAGCATCTCATAACTGCA	Sangon
pD7	5' FAM-GTGACAGCATCTCAATACTGCA	Sangon
**Template strand**	**Sequence (3' to 5')**	
T1-AP	3' CACTGTCGTAGAGTATGAGGX**T**CTCCGATTCCACTTT	Genewiz
T1-AP-A	3' CACTGTCGTAGAGTATGAGGX**A**CTCCGATTCCACTTT	Genewiz
T1-AP-G	3' CACTGTCGTAGAGTATGAGGX**G**CTCCGATTCCACTTT	Genewiz
T1-AP-C	3' CACTGTCGTAGAGTATGAGGX**C**CTCCGATTCCACTTT	Genewiz
tS0	3' CACTGTCGTAGAGTATGACGTCGACTAGTCCACTGT	Sangon

tR0	3' CACTGTCGTAGAGTATGACGXTCGACTAGTCCACTGT	Sangon
tR0-C	3' CACTGTCGTAGAGTATGACGXCGACTAGTCCACTGT	Sangon
tR1	3' CACTGTCGTAGAGTATGACXGTCGACTAGTCCACTGT	Sangon
tR2	3' CACTGTCGTAGAGTATGAXCGTCGACTAGTCCACTGT	Sangon
tR3	3' CACTGTCGTAGAGTATGXACGTCGACTAGTCCACTGT	Sangon
tR4	3' CACTGTCGTAGAGTATXGACGTCGACTAGTCCACTGT	Sangon
tR5	3' CACTGTCGTAGAGTAXTGACGTCGACTAGTCCACTGT	Sangon
tR6	3' CACTGTCGTAGAGTXATGACGTCGACTAGTCCACTGT	Sangon
tR7	3' CACTGTCGTAGAGXTATGACGTCGACTAGTCCACTGT	Sangon
**Cloning primers**	**Sequence (5' to 3')**	
Dpo4-Nde I F	GGCCACTcatatgATTGTTCTTTTCGTTGATTTTG	Tsingke
Dpo4-Sal I R	ATTTgtcgacAGTATCGAAGAACTTGTCTAAC	Tsingke

X denotes a synthetic abasic site.

Supplementary Table S2. DNA Substrates Used in This Study

Substrates (primer-template)	Sequence (5' to 3' for top strand and 3' to 5' for bottom strand)
S_{NT} (P2-T1AP, AP-TLS insertion)	GTGACAGCATCTCATACTCC<br>CACTGTCGTAGAGTATGAGGX**T**CTCCGATTCCACTTT
S_{NA} (P2-T1AP-A)	GTGACAGCATCTCATACTCC<br>CACTGTCGTAGAGTATGAGGX**A**CTCCGATTCCACTTT
S_{NG} (P2-T1AP-G)	GTGACAGCATCTCATACTCC<br>CACTGTCGTAGAGTATGAGGX**G**CTCCGATTCCACTTT
S_{NC} (P2-T1AP-C)	GTGACAGCATCTCATACTCC<br>CACTGTCGTAGAGTATGAGGX**C**CTCCGATTCCACTTT
L_{AT} (P3-T1AP, AP-TLS extension)	GTGACAGCATCTCATACTCC**A**<br>CACTGTCGTAGAGTATGAGGX**T**CTCCGATTCCACTTT
L_{TT} (P3T-T1AP)	GTGACAGCATCTCATACTCC**T**<br>CACTGTCGTAGAGTATGAGGX**T**CTCCGATTCCACTTT
L_{GT} (P3G-T1AP)	GTGACAGCATCTCATACTCC**G**<br>CACTGTCGTAGAGTATGAGGX**T**CTCCGATTCCACTTT
L_{CT} (P3C-T1AP)	GTGACAGCATCTCATACTCC**C**<br>CACTGTCGTAGAGTATGAGGX**T**CTCCGATTCCACTTT
L_{AA} (P3-T1AP-A)	GTGACAGCATCTCATACTCC**A**<br>CACTGTCGTAGAGTATGAGGX**A**CTCCGATTCCACTTT
L_{TA} (P3T-T1AP-A)	GTGACAGCATCTCATACTCC**T**<br>CACTGTCGTAGAGTATGAGGX**A**CTCCGATTCCACTTT
L_{GA} (P3G-T1AP-A)	GTGACAGCATCTCATACTCC**G**<br>CACTGTCGTAGAGTATGAGGX**A**CTCCGATTCCACTTT
L_{CA} (P3C-T1AP-A)	GTGACAGCATCTCATACTCC**C**<br>CACTGTCGTAGAGTATGAGGX**A**CTCCGATTCCACTTT
L_{AG} (P3-T1AP-G)	GTGACAGCATCTCATACTCC**A**<br>CACTGTCGTAGAGTATGAGGX**G**CTCCGATTCCACTTT
L_{TG} (P3T-T1AP-G)	GTGACAGCATCTCATACTCC**T**<br>CACTGTCGTAGAGTATGAGGX**G**CTCCGATTCCACTTT
L_{GG} (P3G-T1AP-G)	GTGACAGCATCTCATACTCC**G**<br>CACTGTCGTAGAGTATGAGGX**G**CTCCGATTCCACTTT

L_{CG} (P3C-T1AP-G)	GTGACAGCATCTCATACTCC**C**<br>CACTGTCGTAGAGTATGAGGX**G**CTCCGATTCCACTTT
L_{AC} (P3-T1AP-C)	GTGACAGCATCTCATACTCC**A**<br>CACTGTCGTAGAGTATGAGGX**C**CTCCGATTCCACTTT
L_{TC} (P3T-T1AP-C)	GTGACAGCATCTCATACTCC**T**<br>CACTGTCGTAGAGTATGAGGX**C**CTCCGATTCCACTTT
L_{GC} (P3G-T1AP-C)	GTGACAGCATCTCATACTCC**G**<br>CACTGTCGTAGAGTATGAGGX**C**CTCCGATTCCACTTT
L_{CC} (P3C-T1AP-C)	GTGACAGCATCTCATACTCCC<br>CACTGTCGTAGAGTATGAGGXCCTCCGATTCCACTTT
D1 (pL0-tR0-C)	GTGACAGCATCTCATACTGC**A**<br>CACTGTCGTAGAGTATGACGXCGACTAGTCCACTGT
D2 (pD1-tR0)	GTGACAGCATCTCATACTGC**AA**<br>CACTGTCGTAGAGTATGACGXTCGACTAGTCCACTGT
D3 (pD2-tR1)	GTGACAGCATCTCATACTG**ACA**<br>CACTGTCGTAGAGTATGACXGTCGACTAGTCCACTGT
D4 (pD3-tR2)	GTGACAGCATCTCATACT**AGCA**<br>CACTGTCGTAGAGTATGAXCGTCGACTAGTCCACTGT
D5 (pD4-tR3)	GTGACAGCATCTCATAC**ATGCA**<br>CACTGTCGTAGAGTATGXACGTCGACTAGTCCACTGT
D6 (pD5-tR4)	GTGACAGCATCTCATA**ACTGCA**<br>CACTGTCGTAGAGTATXGACGTCGACTAGTCCACTGT
D7 (pD6-tR5)	GTGACAGCATCTCAT**AACTGCA**<br>CACTGTCGTAGAGTAXTGACGTCGACTAGTCCACTGT
D8 (pD7-tR6)	GTGACAGCATCTCA**ATACTGCA**<br>CACTGTCGTAGAGTXATGACGTCGACTAGTCCACTGT
S0 (pL0-tS0)	GTGACAGCATCTCATACTGCA<br>CACTGTCGTAGAGTATGACGTCGACTAGTCCACTGT

L1 (pL0-tR0)	GTGACAGCATCTCATACTGC**A**<br>CACTGTCGTAGAGTATGACGXTCGACTAGTCCACTGT
L2 (pL0-tR1)	GTGACAGCATCTCATACTG**CA**<br>CACTGTCGTAGAGTATGACXGTCGACTAGTCCACTGT
L3 (pL0-tR2)	GTGACAGCATCTCATACT**GCA**<br>CACTGTCGTAGAGTATGAXCGTCGACTAGTCCACTGT
L4 (pL0-tR3)	GTGACAGCATCTCATAC**TGCA**<br>CACTGTCGTAGAGTATGXACGTCGACTAGTCCACTGT
L5 (pL0-tR4)	GTGACAGCATCTCATA**CTGCA**<br>CACTGTCGTAGAGTATXGACGTCGACTAGTCCACTGT
L6 (pL0-tR5)	GTGACAGCATCTCAT**ACTGCA**<br>CACTGTCGTAGAGTAXTGACGTCGACTAGTCCACTGT
L7 (pL0-tR6)	GTGACAGCATCTCA**TACTGCA**<br>CACTGTCGTAGAGTXATGACGTCGACTAGTCCACTGT
L8 (pL0-tR7)	GTGACAGCATCTC**ATACTGCA**<br>CACTGTCGTAGAGXTATGACGTCGACTAGTCCACTGT

Supplementary Table S3. Kinetics parameters for primer extension past AP by Dpo2 and Dpo4

Substrates	Sequence	K_m (μM)	k_{cat} (min^{-1})	k_{eff} (min^{-1} $μM^{-1}$)	Relative k_{eff} (%)
**Dpo2**					
S0	GTGACAGCATCTCATACTGCA<br>CACTGTCGTAGAGTATGACGTCGACTAGTCCACTGT	118 ±22.7	31.5 ±7.5	0.27 ±0.07	100
L1	GTGACAGCATCTCATACTGC^A<br>CACTGTCGTAGAGTATGACGXTCGACTAGTCCACTGT	396 ±169	2.40 ±0.52	0.0065 ±0.0015	2.39 ±0.56
L2	GTGACAGCATCTCATACTG^CA<br>CACTGTCGTAGAGTATGACXGTCGACTAGTCCACTGT	84.5 ±27.8	6.04 ±1.8	0.073 ±0.0089	26.8 ±3.28
L3	GTGACAGCATCTCATACT^GCA<br>CACTGTCGTAGAGTATGAXCGTCGACTAGTCCACTGT	88.9 ±37.9	10.0 ±4.1	0.17 ±0.02	42.6 ±7.29
L4	GTGACAGCATCTCATAC^TGCA<br>CACTGTCGTAGAGTATGXACGTCGACTAGTCCACTGT	149 ±32.2	13.0 ±3.0	0.087 ±0.011	32.2 ±3.98
L5	GTGACAGCATCTCATACTGCA<br>CACTGTCGTAGAGTATXGACGTCGACTAGTCCACTGT	227 ±44.8	6.07 ±1.4	0.027 ±0.0028	9.84 ±1.04
L6	GTGACAGCATCTCAT^ACTGCA<br>CACTGTCGTAGAGTAXTGACGTCGACTAGTCCACTGT	84.6 ±17.9	11.8 ±1.3	0.14 ±0.016	52.3 ±5.83
L7	GTGACAGCATCTCA^TACTGCA<br>CACTGTCGTAGAGTXATGACGTCGACTAGTCCACTGT	113 ±33.6	18.4 ±3.7	0.17 ±0.064	64.0 ±23.4
L8	GTGACAGCATCTC^ATACTGCA<br>CACTGTCGTAGAGXTATGACGTCGACTAGTCCACTGT	128 ±41.0	37.8 ±19.9	0.28 ±0.057	104 ±21.1
D1	GTGACAGCATCTCATACTGCA<br>CACTGTCGTAGAGTATGACGXCGACTAGTCCACTGT	835 ±95.4	0.120 ±0.0018	0.00015 ±0.000018	0.0533 ±0.0066
D2	GTGACAGCATCTCATACTGCAA<br>CACTGTCGTAGAGTATGACGXTCGACTAGTCCACTGT	709 ±161	0.58 ±0.20	0.00084 ±0.00026	0.31 ±0.095
D3	GTGACAGCATCTCATACTGACA<br>CACTGTCGTAGAGTATGACXGTCGACTAGTCCACTGT	150 ±46.5	17.3 ±3.7	0.12 ±0.019	43.9 ±7.07
D4	GTGACAGCATCTCATACTAGCA<br>CACTGTCGTAGAGTATGAXCGTCGACTAGTCCACTGT	129 ±38.0	8.12 ±3.4	0.062 ±0.013	22.7 ±4.80
D5	GTGACAGCATCTCATACATGCA<br>CACTGTCGTAGAGTATGXACGTCGACTAGTCCACTGT	245 ±73.3	7.05 ±2.6	0.029 ±0.0067	10.5 ±2.48
D6	GTGACAGCATCTCATAACTGCA<br>CACTGTCGTAGAGTATXGACGTCGACTAGTCCACTGT	166 ±60.8	8.15 ±3.5	0.050 ±0.016	18.3 ±5.89

D7	GTGACAGCATCTCATAACTGCA<br>CACTGTCGTAGAGTAXTGACGTCGACTAGTCCACTGT	136 ±45.4	8.03 ±3.0	0.058 ±0.004	21.5 ±1.46
D8	GTGACAGCATCTCAATACTGCA<br>CACTGTCGTAGAGTXATGACGTCGACTAGTCCACTGT	130 ±33.7	11.4±4.0	0.088 ±0.023	32.6 ±8.47
**Dpo4**					
S0	GTGACAGCATCTCATACTGCA<br>CACTGTCGTAGAGTATGACGTCGACTAGTCCACTGT	103 ±20.7	236 ±147	2.2 ±0.92	100
L1	GTGACAGCATCTCATACTGC^A<br>CACTGTCGTAGAGTATGACGXTCGACTAGTCCACTGT	670 ±145	2.14 ±1.0	0.0031 ±0.0011	0.14 ±0.052
L2	GTGACAGCATCTCATACTG^CA<br>CACTGTCGTAGAGTATGACXGTCGACTAGTCCACTGT	266 ±61.5	21.0 ±8.3	0.078 ±0.019	3.58 ±0.89
L3	GTGACAGCATCTCATACT^GCA<br>CACTGTCGTAGAGTATGAXCGTCGACTAGTCCACTGT	229 ±14.6	15.9 ±1.8	0.069 ±0.0068	3.18 ±0.31
L4	GTGACAGCATCTCATAC^TGCA<br>CACTGTCGTAGAGTATGXACGTCGACTAGTCCACTGT	179 ±51.2	62.0 ±26.8	0.35 ±0.11	16.0 ±5.17
L5	GTGACAGCATCTCATACTGCA<br>CACTGTCGTAGAGTATXGACGTCGACTAGTCCACTGT	66.8 ±7.7	122 ±35.7	1.8 ±0.51	84.0 ±23.3
L6	GTGACAGCATCTCAT^ACTGCA<br>CACTGTCGTAGAGTAXTGACGTCGACTAGTCCACTGT	153 ±33.2	67.4 ±35.2	0.43 ±0.15	19.5 ±6.92
L7	GTGACAGCATCTCA^TACTGCA<br>CACTGTCGTAGAGTXATGACGTCGACTAGTCCACTGT	88.0 ±14.6	170 ±15.6	2.0 ±0.4	90.6 ±18.5
L8	GTGACAGCATCTC^ATACTGCA<br>CACTGTCGTAGAGXTATGACGTCGACTAGTCCACTGT	97.7 ±50.2	208 ±88.7	2.2 ±0.4	102 ±18.3
D1	GTGACAGCATCTCATACTGCA<br>CACTGTCGTAGAGTATGACGXCGACTAGTCCACTGT	451 ±114	2.51 ±1.2	0.0055 ±0.0022	0.25 ±0.10
D2	GTGACAGCATCTCATACTGCAA<br>CACTGTCGTAGAGTATGACGXTCGACTAGTCCACTGT	269 ±35.7	21.3 ±7.4	0.079 ±0.023	3.62 ±1.04
D3	GTGACAGCATCTCATACTGACA<br>CACTGTCGTAGAGTATGACXGTCGACTAGTCCACTGT	257 ±52.8	53.1 ±15.6	0.21 ±0.028	9.39 ±1.26
D4	GTGACAGCATCTCATACTAGCA<br>CACTGTCGTAGAGTATGAXCGTCGACTAGTCCACTGT	158 ±8.3	16.2 ±4.1	0.10 ±0.031	4.75 ±1.44
D5	GTGACAGCATCTCATACATGCA<br>CACTGTCGTAGAGTATGXACGTCGACTAGTCCACTGT	116 ±8.5	68.7 ±35.1	0.58 ±0.26	26.7 ±12.2
D6	GTGACAGCATCTCATAACTGCA<br>CACTGTCGTAGAGTATXGACGTCGACTAGTCCACTGT	253 ±99.2	46.4 ±18.5	0.18 ±0.011	8.38 ±0.52

D7	GTGACAGCATCTCATAACTGCA<br>CACTGTCGTAGAGTAXTGACGTCGACTAGTCCACTGT	125 ±21.4	74.3 ±7.5	0.60 ±0.087	27.6 ±4.0
D8	GTGACAGCATCTCAATACTGCA<br>CACTGTCGTAGAGTXATGACGTCGACTAGTCCACTGT	156 ±51.2	118 ±98.3	0.68 ±0.36	31.4 ±16.7

Relative k_{eff} means the relative replication efficiency (k_{cat}/K_m) of Dpo2 or Dpo4 on AP-containing substrate normalized to that on undamaged control. K_m and k_{cat} values are calculated from three independent experiments and the standard deviation was shown following the ± symbol. The polymerization efficiency (k_{eff}) was examined with dGTP, the next correct incoming nucleotide.

Supplementary Figures

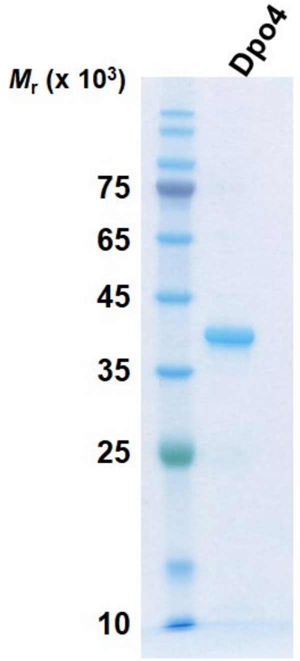

Supplementary Figure S1. SDS-PAGE analysis of purified DNA polymerase from the native host

Purified Dpo4 protein was analyzed by SDS-PAGE and stained with Coomassie Brilliant Blue. The theoretical size of Dpo4 is 40,274 Da.

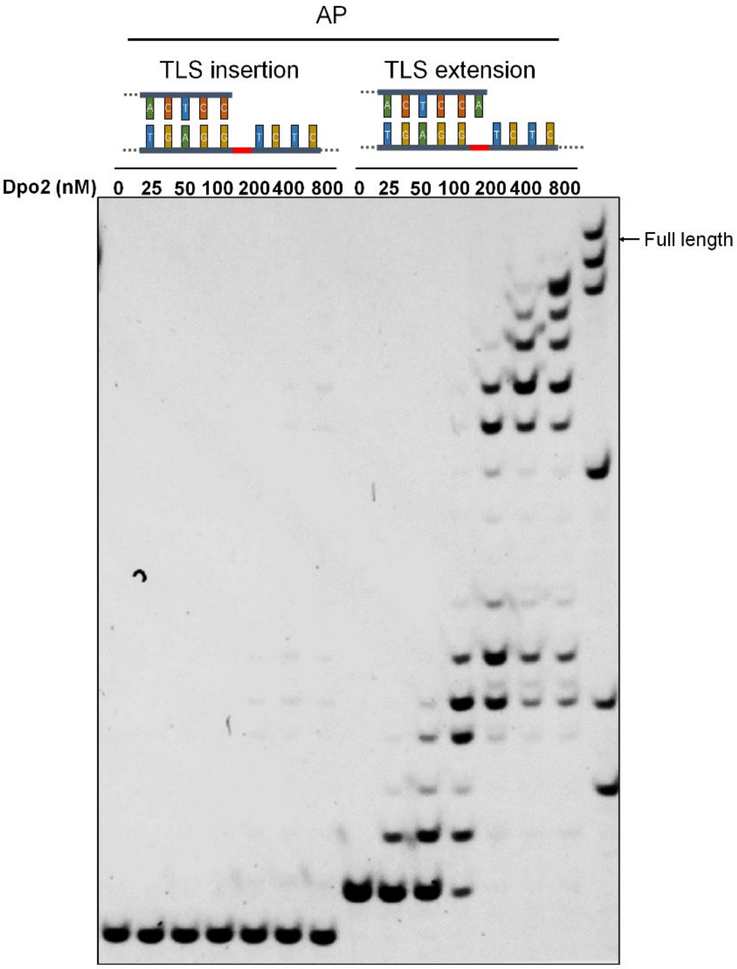

Supplementary Figure S2. TLS extension by *S. islandicus* Dpo2

AP insertion and extension reactions were set up with 100 µM dNTPs and 50 nM S_{NT} (AP insertion) or L_{AT} (AP extension) substrate and the reactions were incubated at 60 °C for 5 min. Dpo2 of different concentrations, as indicated above each lane, were tested. Note that the full length product should be located in the middle of the largest and the second largest marker band based on calculated molecular weights.

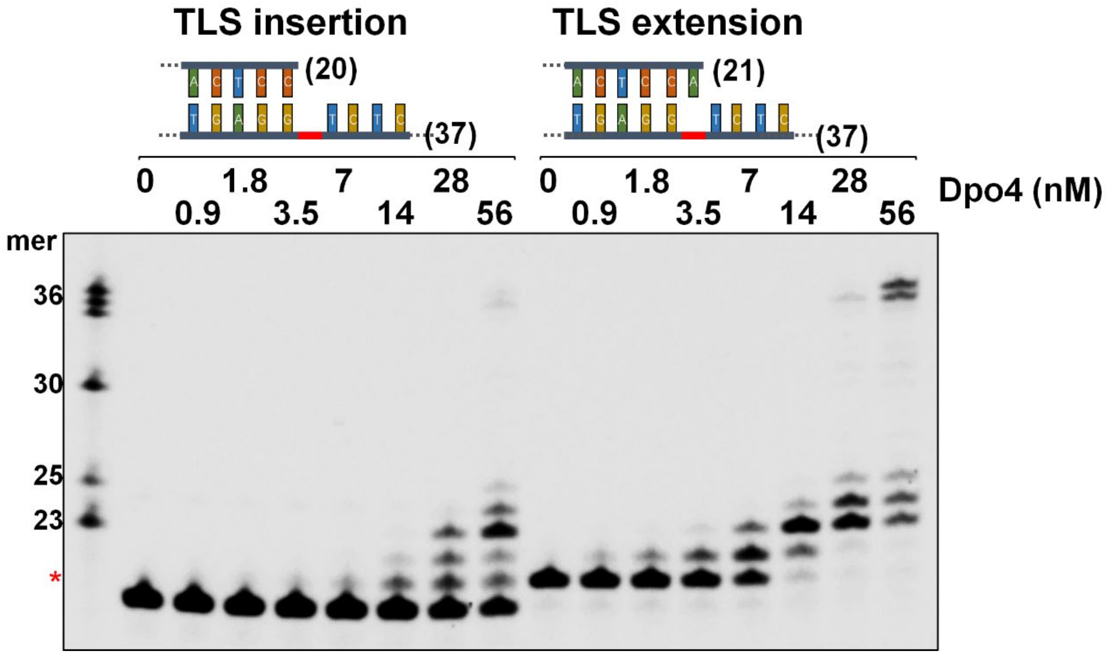

Supplementary Figure S3. The activity of Dpo4 is inhibited past the DNA lesion

TLS insertion and extension assays were set up with 100 µM dNTPs and 50 nM S_{NT} (TLS insertion) or L_{AT} (TLS extension) substrate and the assays were carried out at 60 °C for 5 min. The concentration of Dpo4 used in each reaction was indicated above each lane. The red asterisk symbol indicates the position of AP lesion. Clearly, positions +1, +2 and +3 constitute the inhibitory sites for Dpo4 during the AP extension.

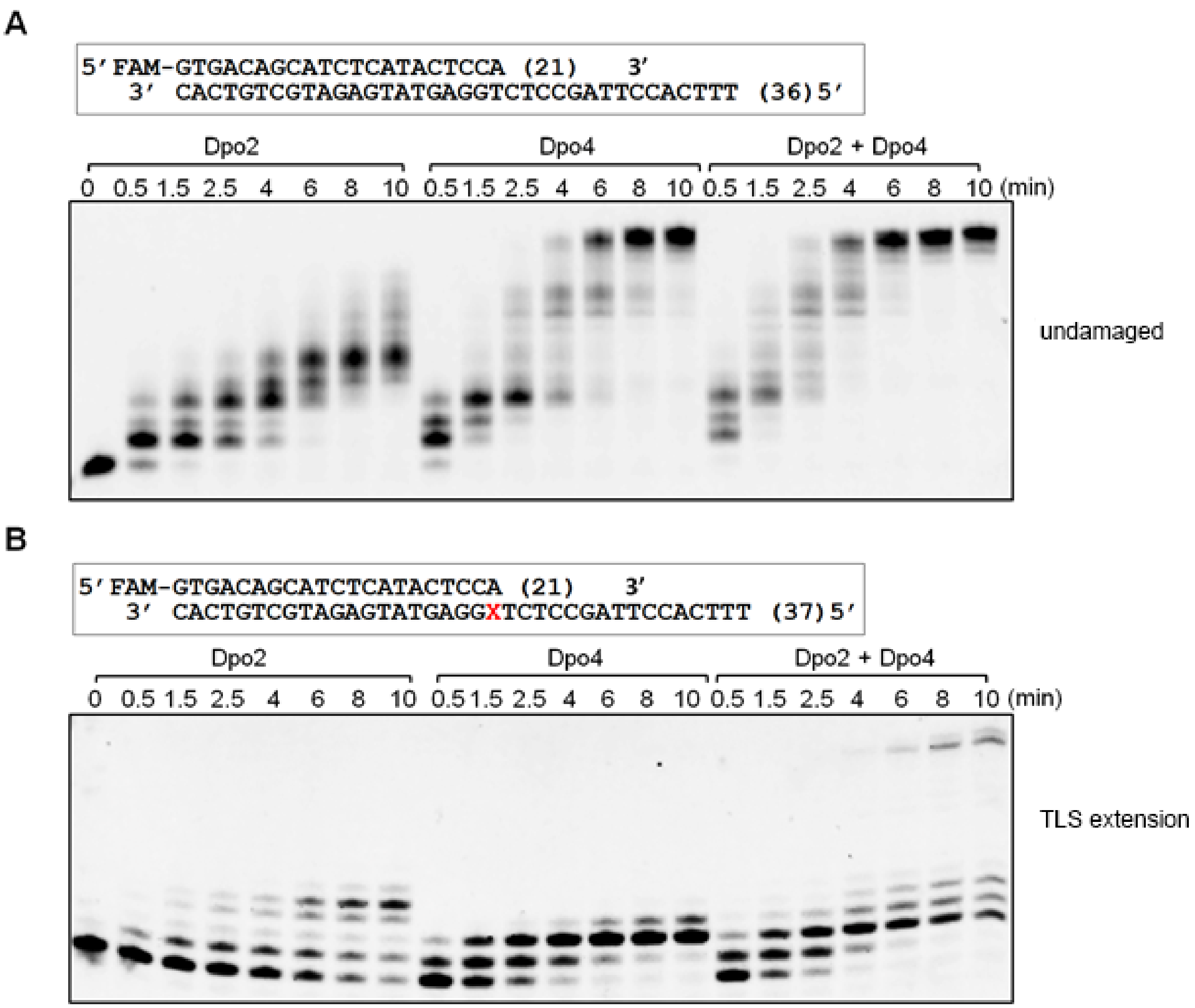

Supplementary Figure S4. Synegestic effect between Dpo2 and Dpo4 was not observed on undamaged substrate

(A) A time course experiment for Dpo2 or Dpo4 on the undamaged template. Each reaction contains 50 nM substrate with sequence indicated above the gel image, 7 nM Dpo4 or 35 nM Dpo2 or both. The assay was conducted at 60 °C for 10 min and samples were taken at indicated time points. (B) A time course experiment for Dpo2 or Dpo4 on the AP-containing template. The X indicates an AP lesion. The experiment was performed as described in panel A.
